# *Bacillus subtilis* Inhibits Viral Hemorrhagic Septicemia Virus Infection in Olive Flounder (*Paralichthys olivaceus*) Intestinal Epithelial Cells

**DOI:** 10.3390/v13010028

**Published:** 2020-12-25

**Authors:** So-Ri Han, Hetron M. Munang’andu, In-Kyu Yeo, Sung-Hyun Kim

**Affiliations:** 1 Fishcare, 7, Pyoseondangpo-ro, Pyoseon-myeon, Seogwipo, Jeju 63629, Korea; Sori_han.fishcare@outlook.kr; 2 Section of Experimental Biomedicine, Department of Production Medicine, Adamstuen Campus, Norwegian University of Life Sciences, P.O. Box 0454, NO-0033 Oslo, Norway; hetroney.mweemba.munangandu@nmbu.no; 3 Department of Marine Life Science, Jeju National University, Jeju 63243, Korea; ikyeo99@jejunu.ac.kr

**Keywords:** *Bacillus subtilis*, surfactin, probiotics, intestinal epithelial cells, VHSV

## Abstract

Viral hemorrhagic septicemia virus (VHSV) is a highly pathogenic virus that infects a wide range of host fish species causing high economic losses in aquaculture. Epithelial cells in mucosal organs are target sites for VHSV entry into fish. To protect fish against VHSV infection, there is a need to develop antiviral compounds able to prevent establishment of infection at portals of virus entry into fish. *Bacillus subtilis* is a probiotic with excellent antiviral properties, of which one of its secretions, surfactin, has been shown to inhibit viral infections in mammals. Herein, we demonstrate its ability to prevent VHSV infection in olive flounder (*Paralichthys olivaceus*) intestinal epithelial cells (IECs) and infection in internal organs. Our findings show inhibition of VHSV infection in IECs by *B. subtilis* and surfactin. In addition, our findings showed inhibition of VHSV in Epithelioma Papulosum Cyprini (EPC) cells inoculated with intestinal homogenates from the fish pretreated with *B. subtilis* by oral exposure, while the untreated fish had cytopathic effects (CPE) caused by VHSV infection in the intestines at 48 h after the VHSV challenge. At 96 h post-challenge, samples from the untreated fish had CPE from head kidney and spleen homogenates and no CPE were observed in the intestinal homogenates, while the *B. subtilis*-pretreated fish had no CPE in all organs. These findings demonstrate that inhibition of VHSV infection at portals of virus entry in the intestines culminated in prevention of infection in internal organs. In summary, our results show that *B. subtilis* has the potential to prevent VHSV infection in fish and that its use as a probiotic in aquaculture has the potential to serve as an antiviral therapeutic agent against different viral infections.

## 1. Introduction

Viral hemorrhagic septicemia virus (VHSV) is an enveloped non-segmented negative-sense single-stranded RNA (ssRNA) virus belonging to the genus *Novirhabdovirus* in the family Rhabdoviridae [1]. It has a wide range of host fish species and has been reported worldwide. Infection of VHSV in farmed olive flounder, *Paralichthys oilvaceus*, was first reported in Korea in 2001 and was later found to be widely distributed in wild olive flounder. Clinical signs and pathology are characterized by exophthalmia, anemia, and distended abdomen due to an edema in the peritoneal cavity. In addition, VHSV causes hemorrhage at the base of the fins, gills, eyes, muscles, and skin. Mortalities reaching greater than 90% have been reported in farmed fish [2], while in wild fish, massive mortalities have been reported in different parts of the world. Several studies have shown that gills and skin are the main portals of virus entry [3,4], while other studies have shown that the gut is also an important entry site for VHSV into fish [5,6]. In these organs, the virus initially attaches to epithelial cells subsequently entering the circulatory system where it is disseminated to internal organs. Thus, epithelial cells generated from various mucosal organs are being used as in vitro models for studying viral entry and virulence mechanisms of VHSV in fish [7,8]. In addition, epithelial cells from different mucosal organs are being used as in vitro models for screening antiviral compounds against viral infections.

Different antiviral compounds have been tested for their ability to inhibit VHSV infection. These include compounds such as JL122, an oxazolidine-2,4-dithione compound [9], olive extracts [10], flavonoid extracts of *Rhus verniciflua* [11], interferon-induced protein with tetratricopeptide repeats 5 (IFIT5) [12], pentacyclic triterpenoids extracted from *Sanguisorba officinalis* roots [13], methanolic extract of *Sanguisorba officinalis* L. roots [14], and algal *Eklonia cava* extracts [15]. While the search for antiviral compounds against VHSV has continued to increase in recent years, an important challenge raising significant concerns is the mode of delivery of these antiviral compounds into fish. Antiviral compounds that require delivery by injection impact stress on fish due to individual handling of fish, while the antiviral compounds delivered by immersion in aquatic environments used for fish farming risk the danger of being taken by other aquatic organisms, in which they might cause adverse effects. Hence, oral delivery of antiviral compounds through feed or probiotics is perceived to be a better alternative, as this is likely not to induce stress in fish and poses a lower risk of excess deposition of antiviral compounds in aquatic environments. Another possible advantage of oral delivery of antiviral compounds through feed is that fewer active compounds would be needed for oral exposure unlike delivery by injection.

An important antiviral compound shown to have potential for oral delivery as a probiotic is surfactin [16,17]. This compound is produced by several bacteria species, of which the most extensively studied are *Pseudomonas aeruginosa*, *Acinetobacter calcoacetic,* and *Bacillus subtilis* [18,19,20,21,22]. Among these, the most widely used as a probiotic in different host species, including fish, is *B. subtilis* [17,23,24]. *B. subtilis* and surfactin have been shown to inhibit infections caused by different viruses, including rhabdoviral infections like vesicular stomatitis virus (VSV) [25,26,27,28]. However, there are no studies reported thus far on inhibition of rhabdoviral infections in fish by *B. subtilis* and surfactin. Hence, in this study, we wanted to evaluate the ability of *B. subtilis* and surfactin to inhibit VHSV infection in olive flounder intestinal epithelial cells (IECs) as a proxy in vitro model for VHSV infection. In addition, we also wanted to determine the ability of *B. subtilis* and surfactin to inhibit VHSV infection in mucosal and internal organs of olive flounder. Overall, we anticipate that the data generated herein will pave way for the use of *B. subtilis* probiotics as an antiviral approach for the prevention of viral infections in aquaculture.

## 2. Materials and Methods

### 2.1. Preparation of Primary Intestinal Epithelial Cells

To develop a proxy in vitro model for VHSV infection, primary IECs from healthy juvenile olive flounder (<15 g) were isolated and cultured using a protocol modified from a previous study [3]. First, intestinal tissues were dissected and put in a solution containing antibiotics (gentamicin, Gibco, ThermoFisher Scientific, Waltham, MA, USA, 10 mg/mL; penicillin-Streptomycin (PEST), Gibco, 10,000 U/mL) and an antifungal (amphotericin B, Gibco, 250 µg/mL). Blood clots in the intestinal tissues were removed followed by trypsinization in 0.5% trypsin–EDTA (Gibco) for 20 min. The trypsin activity was stopped by adding 10% fetal bovine serum (FBS; Gibco) to the phosphate-buffered saline (PBS). IECs were collected and seeded in cell culture flasks in the Gibco Leibovitz’s L-15 Medium (Gibco) containing 10% FBS and 1% of gentamicin and PEST. Thereafter, the flasks were incubated at 20 °C for 24 h. After 24 h, the suspended particles and blood cells in the cell culture medium were removed by washing twice using PBS. The cell culture medium was replaced every second day and the cells were incubated at 20 °C until confluent.

### 2.2. Use of Intestinal Epithelial Cells (IECs) as a Proxy Model VHSV Inhibition

To test the antiviral activity of *B. subtilis* and surfactin against VHSV, we used primary IECs as an in vitro proxy VHSV infection model. The primary IECs were seeded in 96-well culture plates at a density of 10^5^ cells/well. The plates containing cells were pre-incubated with *B. subtilis* or surfactin for 1 h at 20 °C before inoculation of VHSV as described below. Briefly, viable *B. subtilis* (isolation DH2, Daeho, Korea) in L-15 and surfactin (Sigma–Aldrich, St. Louis, MO, USA) in 95% ethanol were added to the inoculum at concentrations of 10^3^ to 10^5^ CFU/mL of *B. subtilis* and 0.1 to 0.3 mg/mL of surfactin, respectively. Wildtype VHSV (JF-09, multiplicity of infection (MOI) = 10) (Kim et al., 2014) was inoculated into the cells 1 h after the *B. subtilis* and surfactin treatment. Cells not treated with *B. subtilis* or surfactin but inoculated with VHSV were used as the positive control. The cells used as the negative control were not inoculated with the virus and they were not treated with *B. subtilis* or surfactin. Three plates were used for each treatment group including the control groups. For viability analysis, the virus-infected cells were incubated for 96 h at 20 °C. The cytopathic effect (CPE) was determined by microscopy examination every 24 h and after 96 h, the results were obtained using the the 3-(4,5-dimethylthiazol-2-yl)-2,5-diphenyl-2H-tetrazolium bromide (MTT) assay [29] that measures cellular growth and survival by detecting only the living cells. The absorbance was read at 490 nm using an Epoch Microplate Spectrophotometer (Gen5 3.03, BioTek Instruments, Winooski, VT, USA).

### 2.3. Bacillus Subtilis Inhibitory Tests against VHSV Infection in Olive Flounder

*B. subtilis* was tested to determine its inhibitory effect against VHSV infection in olive flounder. To do this, a total of 90 juvenile olive flounder (50 ± 5 g) were divided into three groups with each group having 30 fish. Each group was subdivided into three replicates cultured at 17 °C. The groups consisted of (i) group I: *B. subtilis*-pretreated fish infected with VHSV; (ii) group II: *B. subtilis*-untreated fish infected with VHSV; and (iii) group III: *B. subtilis*-untreated fish not infected with VHSV. The *B. subtilis*-pretreated fish infected with VHSV (group I) were orally inoculated with 0.1 ml/fish of 10^5^ CFU/mL of *B. subtilis* using a syringe. This was repeated three times at intervals of 30 min and was completed 72 h before VHSV inoculation. Groups II and III were orally inoculated with PBS three times at intervals of 30 min and completed 72 h before VHSV inoculation. Seventy-two hours after group I was treated with *B. subtilis*, the pretreated (group I) and untreated infected (group II) groups were orally exposed to VHSV at a concentration of 10^6^ tissue culture infective dose 50 per mL (TCID_50_/mL) using syringes. At 48 and 96 h after the VHSV infection, three fish from each group were sampled for the detection of VHSV by cell culture and PCR as described below. The tissues collected included the intestine, spleen, and kidney. The tissue samples were homogenized separately in L-15 growth media containing gentamicin, Gibco, 10 mg/mL; PEST, Gibco, 10,000 U/mL, and an antifungal (amphotericin B, Gibco, 250 µg/mL). The homogenates were filtered through a 0.2 µm syringe filter. The filtered liquids were inoculated onto EPC (Epithelioma Papulosum Cyprini) (ATCC^®^ CRL-2872^™^) cells in 24-well plates. At 72 h post-inoculation, the cell culture supernatants were passaged for the second time into new EPC confluent cells in 24-well plates. The cytopathic effect (CPE) was determined by microscopy examination every 24 h and the final examination after 168 h.

The CPEs caused by VHSV infections were confirmed by PCR using the RNA extracted from infected EPC cells, while non-infected cells were used as negative controls. RNA extraction and cDNA synthesis together with PCR reactions were carried out as previously described by Nishizawa et al. [30] using the forward primer 5′-CCAGCTCAACTCAGGTGTCC-3′ and the reverse primer 5′-GTCACYGTGCATGCCATTGT3′. Gel electrophoresis analysis was carried to determine the size of the PCR products from infected EPC cells in comparison with the positive control VHSV samples.

The Student’s t-test was used to determine the level of significance of the difference between the groups, while the Pearson correlation coefficients were used to determine the correlation between the increase in *B. subtilis* or surfactin concentration and inhibition of VHSV in the IEC cells.

## 3. Results

### 3.1. Primary Intestinal Epithelial Cells

**Figure 1:**
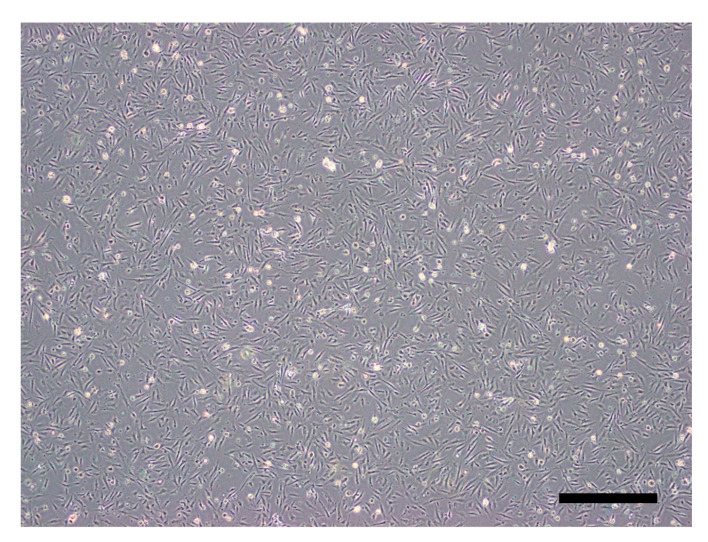
Olive flounder intestinal epithelial cells (IECs) visualized by phase-contrast microscopy at 96 h post-seeding (40× magnification) (scale bar = 500 μm).

Primary IECs derived from olive flounder intestinal tissues were confluent after culturing at 20 °C for four days (Figure 1).

### 3.2. MTT Assay Results

**Figure 2:**
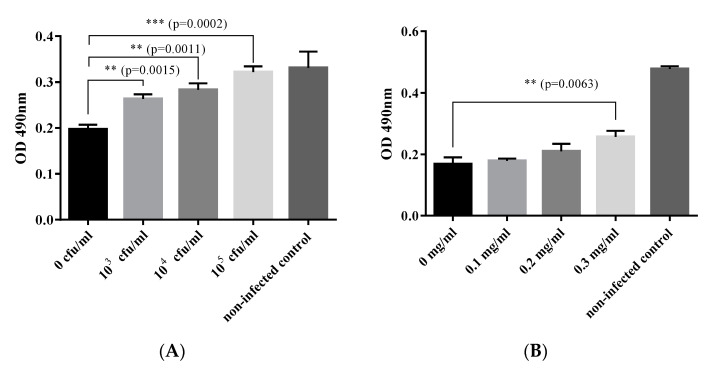
Inhibition of viral hemorrhagic septicemia virus (VHSV) (MOI = 10) infection of olive flounder by *B. subtilis* (**A**) and surfactin (**B**) determined by the MTT assay (bar chart shows means with the SD, *n* = 3 plates). ** denote significance level ≤0.002 while *** denote significance level ≤0.0002.

The ability of *B. subtilis* and surfactin to inhibit CPE induced by VHSV was demonstrated in primary IECs (Figure 2). Note that *B. subtilis* inhibited CPE formation significantly stronger (*p* = 0.0015) in the 10^3^ CFU/mL pretreated cells than in the untreated cells. Note also that the strongest inhibition of CPE was in the IECs exposed to the highest *B. subtilis* concentration of 10^5^ CFU/mL (Figure 2A) having the most potent inhibitory activity (*p* = 0.0002). The inhibition of CPE in *B. subtilis*-pretreated cells inoculated with VHSV increased with the corresponding increase of the *B. subtilis* concentration added to the cells (Figure 2A). We found a high positive correlation (r^2^ = 0.973, *p* = 0.0001) between the inhibition of CPE and increase of the *B. subtilis* concentration in the primary IECs inoculated with VHSV. The inhibition of CPE in VHSV-inoculated IECs induced by surfactin was insignificantly low for 0.1 mg/mL and 0.2 mg/mL concentrations (Figure 2B). However, surfactin significantly (*p* = 0.0063) inhibited CPE formation in VHSV-inoculated cells at 0.3 mg/mL. The ability of ethanol to inhibit VHSV infection using similar concentrations with surfactin was tested but showed no inhibitory effect (data not shown). Overall, our findings show that both *B. subtilis* and surfactin inhibited CPE formation in the primary IECs inoculated with VHSV.

### 3.3. Inhibitory Tests against VHSV in Olive Flounder

**Figure 3:**
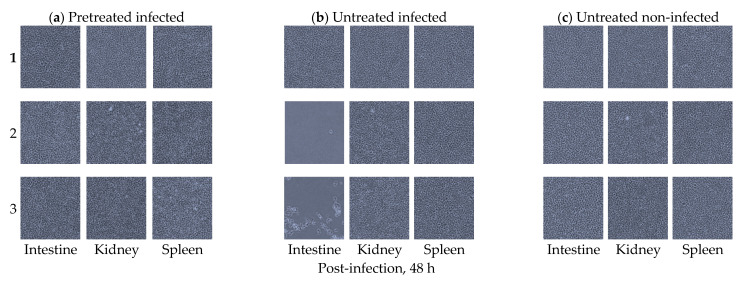
EPC cells inoculated with intestine, kidney, or spleen tissue homogenates from olive flounder (*Paralichthys olivaceus*). (**a**) Replicates of intestine, kidney, and spleen tissue homogenates cultured in EPC cells. Homogenates are from the orally *B. subtilis*-pretreated olive flounder at a concentration of 10^6^ CFU/mL examined 48 h after the VHSV infection. Note the absence of cytopathic effects (CPE) in all the tissues examined. (**b**) Replicates of intestine, kidney, and spleen tissue homogenates cultured in EPC cells. Homogenates are from *B. subtilis*-untreated olive flounder examined 48 h after the VHSV infection. Note the presence of CPE in two samples of the intestines and absence of CPE in the spleen and kidney replicates. (**c**) Replicates of intestine, kidney, and spleen homogenate tissues cultured in EPC cells from *B. subtilis*-untreated olive flounder that were not infected by VHSV examined after 48 h. Note the absence of CPE in all the tissues examined. All images were visualized by phase-contrast microscopy at 100× magnification.

Inhibitory effects of *B. subtilis* against VHSV infection in olive flounder were demonstrated in the EPC cells inoculated with homogenates from intestine, kidney, and spleen samples collected 48 h after the oral VSHV infection (Figure 3).

**Figure 4:**
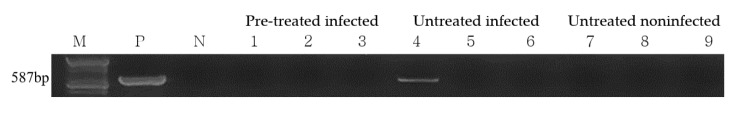
PCR analysis of the EPC cells inoculated with intestine, kidney, or spleen tissue homogenates from olive flounder (*Paralichthys olivaceus*). Lanes 1, 4, and 7 = intestine; lanes 2, 5, and 8 = kidney; lanes 3, 6, and 9 = spleen; N = negative control (deionized sterile water); P = positive control (VHSV); M: 100 bp DNA ladder.

Note that the *B. subtilis*-pretreated group I had no CPE in all replicate EPC monolayers inoculated with intestine, kidney, and spleen homogenates being similar with the observations made in the *B. subtilis*-untreated fish not exposed to VHSV (group III). On the contrary, EPC cells inoculated with intestine homogenates from the *B. subtilis*-untreated fish that were orally infected by VHSV (group II) had CPE indicating the intestines had a viable virus. However, the EPC cells inoculated with kidney and spleen homogenates had no CPE in all the three groups (groups I to III) indicating that there was no virus in these organs at 48 h after the oral VHSV exposure. All the samples showing CPE were subjected to PCR analysis using the primers described in Section 2.3 above followed by gel electrophoresis analysis. Positive PCR bands from CPE-positive EPC cells inoculated with pooled intestine samples were detected like in the VHSV-positive control sample (untreated infected group II), but no bands were detected in the negative control non-infected cells and the deionized water control sample (Figure 4).

**Figure 5:**
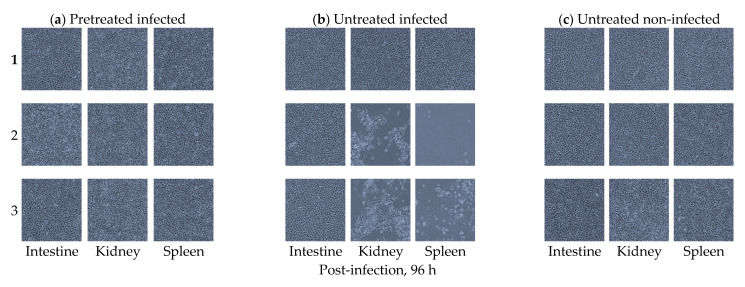
Intestinal epithelial cells (IECs), intestine, kidney, and spleen tissues cultured from olive flounder (*Paralichthys olivaceus*). (**a**) Replicates of intestine, kidney, and spleen homogenates cultured in EPC cells from the orally *B. subtilis*-pretreated olive flounder at a concentration of 10^6^ CFU/mL examined 96 h after the VHSV infection. Note the absence of cytopathic effects (CPE) in all the tissues examined. (**b**) Replicates of intestine, kidney, and spleen homogenates cultured in EPCs from the *B. subtilis*-untreated olive flounder examined 96 h after the VHSV infection. Note the presence of CPE in two cases of the EPC cells inoculated with spleen and kidney samples and the absence of CPE in all intestine replicates. (**c**) Replicates of intestine, kidney, and spleen homogenates cultured in EPC cells from *B. subtilis*-untreated olive flounder that were not infected by VHSV examined after 96 h. Note the absence of CPE in all the tissues examined. All images were visualized by phase-contrast microscopy at 100× magnification.

Inhibitory effects of *B. subtilis* against VHSV infection in olive flounder were shown in the EPC cells inoculated with tissue homogenates from the intestine, kidney, and spleen samples collected from olive flounder at 96 h after the oral VHSV exposure (Figure 5).

**Figure 6:**
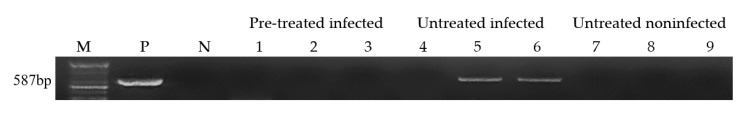
PCR analysis of the EPC cells inoculated with intestine, kidney, or spleen tissue homogenates from olive flounder (*Paralichthys olivaceus*). Lanes 1, 4, and 7= intestine; lanes 2, 5, and 8 = kidney; lanes 3, 6, and 9 = spleen; N = negative control (deionized sterile water); P = positive control (VHSV); M: 100 bp DNA ladder.

Note there was no CPE observed in the EPC cells inoculated with tissue homogenates from all the organs collected from the *B. subtilis*-pretreated group (group I) at 96 h after the oral VSHV exposure similar to the EPC cells inoculated with tissue homogenates from the non-infected control group (group III). On the contrary, CPE was observed in two wells inoculated with kidney tissue homogenates and in two wells with spleen tissue homogenates from the *B. subtilis*-untreated fish that were infected with VHSV (group II). All the samples were subjected to PCR analysis using the primers described in Section 2.3 above followed by gel electrophoresis analysis. Positive PCR bands from the pooled CPE-positive EPC cells inoculated with kidney and spleen samples were detected in the VHSV-positive control samples (group II (untreated infected group)), but no bands were detected in the negative control non-infected cells and the deionized water control sample (Figure 6).

## 4. Discussion

The role of *B. subtilis* as a probiotic able to improve nutrient assimilation in fish has been reported by several scientists [23,31]. Despite this, its role as an antiviral compound against fish viruses has not been widely investigated as done for mammalian viruses [17,24]. In mammals, Vollenbroich et al. [24] tested the ability of the *B subtilis*-derived surfactin to inhibit several viruses including VSV, Semliki Forest virus (SFV), herpes simplex virus (HSV-1, HSV-2), Suid herpesvirus (SHV-1), simian immunodeficiency virus (SIV), feline calicivirus (FCV), murine encephalomyocarditis virus (EMCV) and showed that enveloped viruses were more susceptible to surfactin inhibition than non-enveloped viruses. Yuan et al. [17] showed that the *B. subtilis*-derived surfactin suppressed the proliferation of porcine epidemic diarrhea virus (PEDV) and transmissible gastroenteritis virus (TGEV) in epithelial cells at a relatively low concentration ranging from 15 to 50 μg/mL without causing cell cytotoxicity. These findings indicate that the *B. subtilis*-derived surfactin is a highly potent antiviral compound able to inhibit viral infections at low concentrations that do not cause cytotoxicity to host cells. Similarly, we found significant inhibition of VHSV, which is an enveloped virus, at low *B. subtilis* (10^3^ to 10^5^ CFU/mL) and surfactin (0.3 mg/mL) concentrations in the present study.

Wang et al. [27] showed that *B. subtilis* OKB105 and surfactin effectively inhibited TGEV from entering porcine intestinal epithelial cells (IPEC-J2) in a dose-dependent manner. Similarly, Vollenbroich et al. [24] showed concentration-dependent inhibition of SHV-1, HSV-1, HSV-2, VSV, SIV, and SFV cultured in different cell lines. They found that the inactivation rate increased logarithmically in a linear trend that corresponded with the increase of the surfactin concentration. They noted that doubling the concentration from 25 to 50 µM resulted in an increase of the inactivation rate by a factor of 1.4 for some viruses [24]. In the present study, we found a significant high correlation (r^2^ = 0.973, *p* < 0.001) between the inhibition of VHSV and the increasing concentration of *B. subtilis* treatment in IECs. However, surfactin only showed significant inhibition of VHSV in IECs at a concentration of 0.3 mg/mL. Taken together, these findings show that *B. subtilis* and surfactin have antiviral properties capable of inhibiting VHSV and other viruses infecting different host species [24,27].

Several studies show that mucosal organs get exposed to viral pathogens earlier than internal organs because of their direct contact with the exterior environment [3,4,5,6]. Therefore, it is likely that mucosal organs could get infected earlier than internal organs that get exposed to the virus after dispersal following initial replication at portals of entry in mucosal organs. Hence, our observation of CPE in the intestine homogenates inoculated on EPC cells at 48 h after the VHSV exposure could be due to the deposition of the virus or early infection in the intestine as a portal of virus entry into fish. On the contrary, no CPE was detected in the EPCs inoculated with kidney and spleen (note that these are internal organs) samples at 48 h after the VHSV exposure as this required the virus to be dispersed to these organs after entry and replication in the intestinal mucosa. In addition, no positive PCR bands were detected in the EPCs inoculated with kidney and spleen samples. Hence, it is likely that by the time the spleen and kidneys had the virus capable of produceing CPE at 96 h after the VHSV exposure, intestinal tissues at portals of virus entry had recovered. Thus, no CPE was detected in intestine samples at 96 h after the VHSV exposure when CPE was observed in the EPCs inoculated with kidney and spleen samples. However, the absence of CPE in the *B. subtilis*-pretreated group suggests that fish that were protected against VHSV infection by the antiviral *B. subtilis* effect at portals of virus entry in the intestine had no virus dispersed to internal organs. Hence, they had no CPE in the spleen and kidneys at 96 h after the virus exposure. This was supported by PCR analysis that showed absence of positive PCR bands in the *B. subtilis*-pretreated group. On the contrary, untreated fish that were not exposed to *B. subtilis* had the virus detected in the EPC cells inoculated with kidney and spleen samples at 96 h after the virus exposure. In addition, they had VHSV-positive PCR bands detected by gel electrophoresis analysis. These findings suggest that antiviral *B. subtilis* effects prevent VHSV infection at portals of virus entry in mucosal organs, which could prevent virus dispersal to internal organs. This is in line with Hong et al. [32] who showed that intranasal administration of *B. subtilis* spores resulted in inhibition of respiratory syncytial virus (RSV) infection in the respiratory tract, which resulted in preventing the pathology in the lungs. Similarly, Yuan et al. [17] showed that oral administering of the *B. subtilis*-derived surfactin protected piglets against PEDV infection, while Canning et al. [33] showed insignificantly weak pathological changes in the piglets treated with *B. subtilis* unlike the untreated piglets that had severe pathology. Jiang et al. [34] showed that the grass carp (*Ctenopharyngodon idellus*) orally given *B. subtilis* had insignificantly low grass carp reovirus (GCRV) loads (almost undetectable levels in the intestines after the challenge), which was linked to insignificantly low viral loads in internal organs, such as the spleen, kidneys, liver, and muscles. On the contrary, *B. subtilis*-untreated fish had high viral loads in the intestines and internal organs. Altogether, these studies support our observation that mucosal *B. subtilis* treatment prevents establishment of infection at portals of viral entry in mucosal organs, which results in preventing the spread of the virus to internal organs.

In conclusion, we have shown that *B. subtilis* and surfactin have antiviral properties against VHSV in olive flounder. In addition, we have also shown that the inhibitory effects of *B. subtilis* and surfactin on VHSV are dose-dependent and that the probiotic use of *B. subtilis* could serve as a therapeutic agent against viral infections in aquaculture.

## Data Availability

Data sharing not applicable.
